# Effect of water bath‐assisted water extraction on physical and chemical properties of soybean oil body emulsion

**DOI:** 10.1002/fsn3.1921

**Published:** 2020-11-20

**Authors:** Haotian Han, Luping Zhao, Xiaonan Liu, Anmin Guo, Xiangyang Li

**Affiliations:** ^1^ School of Food Science and Engineering Shandong Agricultural University Taian China

**Keywords:** emulsifier, fluidity, oil body, oxidation stability, soybean

## Abstract

Soybean oil body (SOB), rich in polyunsaturated fatty acids and biologically active substances, is used as a natural emulsifier in food processing. In addition, SOB is healthier than synthetic emulsifiers. However, the physical and chemical properties of the SOB emulsion directly affect its application in food processing. In order to study the effect of water bath extraction (WBAE) on SOBs, the effects of WBAE method on the composition of SOBs, the zeta potential, average particle size, oxidation stability, and viscosity characteristics of SOB emulsions were researched. It was found that both protein and moisture contents of SOB decreased with increasing WBAE temperature; however, lipid content increased. These results were attributed to the exogenous proteins gradually denatured and dissociated with extraction temperature from 60°C to 100°C. Increasing the extraction temperature, the average particle size of the SOB emulsions increased, the oxidative stability was improved, the Zeta potential and viscosity decreased, and the fluidity of emulsions was improved. The SOB extracted at 100°C has broad application prospects in food, and this research is meaningful for supplying fundamental information for selecting proper extraction temperature of SOBs.

## Graphical abstract

In order to study the effect of water bath extraction (WBE) on soybean oil bodies, the effects of WBE methods on the composition of soybean oil bodies and the zeta potential, average particle size, oxidation stability, and viscosity characteristics of soybean oil body emulsions were studied. The results show that the soybean oil body obtained by WBE has broad application prospects in food.

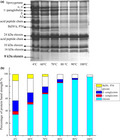

## 1 INTRODUCTION

Soybean, one of the important food crops, has high nutritional value and high protein and oil content and is also the world's most important oil crops (Hartman et al., 2011). Oil bodies, also known as lipid bodies, are subcellular organelle particles that store lipid substances. Most plant seeds store lipid substances in oil bodies (Nikiforidis, 2019; Dave et al., 2019). The oil body has a special structure, the inside is triglyceride, and a single layer of phospholipid membrane is wrapped on the outside, and a small amount of protein (also called endogenous protein) is embedded on the membrane, so the oil body is a natural microcapsule (Tzen, 1992). Soybean oil body (SOB) is rich in fat‐soluble biologically active substances (such as phospholipids, tocopherols, isoflavones) and polyunsaturated fatty acids that are beneficial to human health (Chen, Cao et al., 2014; Chen, Zhao et al., 2014; Fisk & Gray, 2011; Iwanaga et al., 2007). Owing to the special structure, functional components, and good stability characteristics, oil body was used as a natural emulsifier; thus, oil body has broad application prospects in the food, beauty, and medical care industries (Ishii et al., 2017).

At present, different extraction methods and processes of SOB were reported. Tzen et al. (1997) proposed a traditional method for extracting vegetable oil bodies, that is, extracting with an organic solvent, then centrifuging to remove the precipitate, and then washing with detergent, and separating liquid treatment and other steps. Jacks et al. (1990) discovered the method of extracting SOB with water. The water extraction method does not require a homogenous process for extracting SOB, nor an emulsifier, and is nontoxic and harmless, avoiding the extraction of vegetable oils with solvents. Chen et al. (2012) used a two‐step water extraction method to extract more oil bodies. Kapchie et al. (2010) used enzyme‐assisted extraction of SOB and studied the effects of ultrasonic and high static pressure pretreatment methods on oil bodies. The recovery rate of oil bodies extracted by this method is high, up to 84.65%. Not only that, Kapchie and others also conducted a pilot‐scale enzyme‐assisted extraction of SOB, which laid the foundation for the large‐scale industrial production and wide application of SOB.

The extraction methods affected the physicochemical properties and application of SOB. Chen and Ono (2010) obtained SOB at pH 11.0, and the SOB showed good dispersion stability against salt and thermal treatment. However, the SOB extracted at pH 8.6 showed worse dispersion stability (Iwanaga et al., 2007). Zhao et al. (2016) recovered SOB from soybean aqueous extract at different pHs (6.8–11.0). It was found that particle size and viscosity decreased, whereas isoelectric point and oxidative stability increased with increasing extraction pH. Then, SOB extracted at pH 11.0 could be applied to liquid‐type SOB product, and SOB extracted at pH 6.8 could be applied to solid‐type SOB product. As we all know, the biggest advantage of water bath heating is that the temperature can be accurately controlled and the reactants are heated evenly. Then, will the water bath heating‐assisted water extraction process affect the physical and chemical properties of SOB? Will it improve the stability and fluidity of SOB emulsion? Although it has been reported that SOB can be used as emulsifier in food processing, the application of SOB in food is still in its infancy. However, no researches have systematically examined the physicochemical properties and the nutritional composition of SOB obtained from WBAE. The effect of WBAE on the properties of SOB emulsion was discussed, which provided a reference for the application of SOB in food.

## 2 MATERIALS AND METHODS

### 2.1 Materials and instruments

Soybean, Heilongjiang high‐oil soybean. Reduced iron powder, thiobarbituric acid, sodium azide, Tris, sodium acetate, bromophenol blue, coomassie brilliant blue, etc., analytically pure. Acrylamide, N,N'‐k‐omethylenebisacrylamide etc., excellent grade pure, Sigma‐Aldrich company.

Centrifuge 5,804 R refrigerated centrifuge, Ebende AG. Rapid N Cube Rapid Nitrogen Analyzer, Element Analysis System. N‐1001 Rotary Evaporator, Shanghai Ailang Instrument Co., Ltd. MODEL BE‐210 vertical electrophoresis instrument, BIO CRAFT company. ChemiDoc MP gel imager, Bio‐Rad. ZetaPlus Zeta Potentiometer, Brookhaven Instruments.

### 2.2 Methods

#### 2.2.1 Preparation of raw soymilk

First, 50 g soybean seeds were weighed, placed in clean water, and washed three times to remove impurities on the surface of the seeds, and then rinsed with deionized water. The washed seeds were soaked in deionized water in a refrigerator at 4°C for 18 hr. Second, the fully swollen seeds were taken out and put into a tissue masher, adding 4°C fresh deionized water equivalent to 9 times the weight of the weighed soybeans, and beating at a speed of 18,000 r/min for 90 s. Finally, the slurry was filtered with four layers of defatted gauze, and the filtrate (about 600 g) was obtained as raw soymilk.

#### 2.2.2 SOB enrichment extracted at different temperatures

With reference to Cao et al., (2015) and Wu, Huang, et al., (2012); Wu, Yang, et al., (2012), the extraction method of SOB was slightly modified. The SOBs were extracted according to the following methods: The filtered raw soy milk is immediately divided into six parts, one of which was used as a control group and placed in a 4°C refrigerator. The remaining five parts, as the test groups, were heated in water baths of 60°C, 70°C, 80°C, 90°C, and 100°C for 30 min and stirred slowly each 5 min for maintaining uniform heating. Then, the heated soy milk was cooled to room temperature in an ice water bath. Sucrose (20%, w/w) was added to the control group and the test groups and mixed well. All samples were transferred to six 50‐ml centrifuges tube and centrifuged (12,000 r/min, 60 min, 4°C). The floating fractions were collected and were dispersed again into 20% (w/w) sucrose solution (4°C) and centrifuged (12,000 r/min, 60 min, 4°C). The procedure above was repeated twice. The collected floating fractions were SOB enrichments. These SOB enrichments are stored at 4°C for use (the storage time should not exceed 2 days).

#### 2.2.3 Determination of SOB enrichment composition

Moisture content was conducted by oven‐drying with modification. In order to avoid lipid oxidation, the oven was changed into vacuum drying oven, and the temperature was controlled at 90°C.

Protein content was determined by Dumas combustion method, which was used to determine the nitrogen content of the sample.

The lipid content was measured by chloroform–methanol extraction (da Silva Gorg & Aranda, 2013). Each SOB enrichment (1 ~ 2 g) was added into 20 ml chloroform–methanol solution (v/v = 2/1), and the mixture was stirred for 3 hr. Then, the mixture was filtered, and the residue was washed three times with chloroform–methanol solution. The collected filtrate was transferred to a separator funnel, and 0.74% (w/w) NaCl was added into the separator funnel (filtrate/NaCl solution, 5/1, v/v), and the mixture was allowed to separate into layers. The lower phase (containing lipids) was collected and treated by rotary evaporator at 50°C to evaporate organic solvent, further vacuum‐drying at 60°C for 4 hr. The difference weights of the rotary evaporator were the lipid content.

#### 2.2.4 Tricine‐SDS‐PAGE electrophoresis to characterize protein components

Samples were prepared according to the method by Ying et al. (2015). SOBs were mixed with sample dissolution solution in a 2‐ml centrifuge tube and added 10 µl of 0.02% bromophenol blue solution and 20 µl mercaptoethanol into the centrifuge tube. The mixture was heated for 5 min and centrifuged at 10,000 r/min for 10 min. The supernatant was collected.

Tricine‐SDS‐PAGE gels were conducted according to the method by Schägger (2006). The concentrations of stacking and separating acrylamide gels were 4% and 16%, respectively. Each sample (10 μl) was loaded into a sample well, and the samples were electrophoresed at constant voltage of 100 mV until end. After electrophoresis, the gel was fixed with a solution of methyl alcohol/acetic acid (5/1, v/v) for 1 hr. After fixing, gel was stained with 0.025% (w/v) Coomassie Blue G‐250 in 10% (v/v) acetic acid for 2 hr, and gel was destained by 10% (v/v) acetic acid. The band was photographed with a gel imager, and the intensity of the protein band was analyzed with Image Lab 3.0.

#### 2.2.5 Configuration of SOB emulsion

Taking 10% (w/w) SOB emulsion as an example, the configuration method is as follows: 5.0 g (dry weight) of SOB enrichments in the control group and five test groups was weighed, dispersed in 45.0 g deionized water, and stirred with a magnetic stirrer for 10 min to distribute the emulsion. To prevent microbial contamination, 3 mmol/L sodium azide was added to the emulsions, and then, the emulsions were transferred to a 50‐ml glass sample bottle for use.

#### 2.2.6 Zeta potential measurement of SOB emulsion

1% (w/w) SOB emulsions were prepared with control group and five test groups of SOB enrichments, and each emulsion was diluted 400‐fold with phosphate buffer (pH 7.0). At 25°C, the zeta potential of the emulsions was measured by a zeta plus model zeta potentiometer.

#### 2.2.7 Determination of average particle size of SOB emulsion

1% (w/w) SOB emulsions were prepared with control group and five test groups of SOB enrichments, and each emulsion was divided into eight groups on average, and the pH of them was adjusted to 3.0, 4.0, 4.5, 5.0, 5.5, 6.0, 7.0, and 8.0. All samples were diluted 400 times with phosphate buffer solution of the same pH value as the emulsion. At 25°C, the average particle size of the emulsion was measured by a Zeta Plus zeta potentiometer.

The pHs of 1% (w/w) SOB emulsions were adjusted to 8.0, and the samples were diluted 10 times with 30 Mm Tris‐HCl (pH 8.0), and then, the particle size of emulsion was observed by fluorescence inversion microscope.

#### 2.2.8 Determination of oxidation stability of SOB emulsion

The 10% (w/w) SOB emulsion of each group was stored at 37°C for 14 days (with restricted lighting). During storage, 2 ml samples were collected at 0, 1, 2, 5, 8, 11, and 14 days, and the peroxide value and thiobarbituric acid value were measured to monitor the lipid oxidation in the emulsions.

The peroxide value (POV value) was measured according to the method of Manzocco et al. (2020) with some modifications. Each emulsion (0.3 ml) was added into 2‐ml centrifuge tube, and 1.5 ml isooctane isopropanol solution (v/v = 2/1) was added. The centrifuge tube was shaken each 10 s, and the samples were centrifuged for 10 min at 2000 r/min. 0.2 ml supernatant was taken out, and 20 μl of 3.94 mol/L KSCN solution and 20 μl of 0.072 mol/L Fe^2+^ solution were added to the supernatant. It was fixed to 10 ml with methanol‐n‐butanol solution (v/v = 2/1), and the solution was vortexed, and after 20 min (with restricted lighting), the absorbance was measured at 510 nm. The peroxide value is calculated according to the standard curve:

(1) $$\text{POV}\;\left(\text{mmol}/\text{kg}\right)=\left(A_{510}\times \;M\right)/\left(55.84\times 2\times m_{0}\right)$$

where A_510_ is the absorbance at 510 nm; M is the slope of the Fe^3+^ standard curve; m_0_ is the equivalent sample mass when colorimetric, g

Thiobarbituric acid value (TBARS value) was were evaluated by the methods of Yang et al., (2015) and Khouryieh et al. (2015). Each emulsions (3 ml) was mixed with 1% (w/v) thiobarbituric acid solution (2 ml) and 10% (w/v) trichloroacetic acid solution (5 ml), followed by boiling for 30 min and cooling to room temperature. Five ml supernatant was collected in a 15‐ml centrifuge tube and mixed with 5 ml chloroform. The mixture was centrifuged at 4,500 r/min for 20 min. The absorbance value of samples was measured at 532 nm, and the thiobarbituric acid value was calculated as follows:

(2) $$\text{TBARS}\;\left(\text{mg}/L\right)=\left(A_{532}/V\right)\times 9.48$$

where A_532_ is the absorbance at 532 nm; V is the sample volume, ml; and 9.48 is a constant obtained by conversion of molar extinction coefficient.

#### 2.2.9 Determination of viscosity characteristics of SOB emulsion

40% (w/w) SOB emulsions were prepared. The emulsions were placed on the measurement tray of the modular intelligent rheometer, and the viscosity change of SOB emulsion with shear rate was measured by a controlled‐stress rheometer AR1000 with parallel plate geometry (PP50, 50 mm diameter and 1 mm gap). The temperature was regulated by a circulating bath and kept constant at 25°C.

### 2.3 Data analysis and drawing

Each experiment was repeated three times, taking the average value, using Excel 2007, SPSS 23.0, origin 8.0, design‐expert 8.6 for data analysis and drawing.

## 3 RESULTS AND DISCUSSION

### 3.1 Effect of WBAE on the composition of SOB enrichment

**FIGURE 1:**
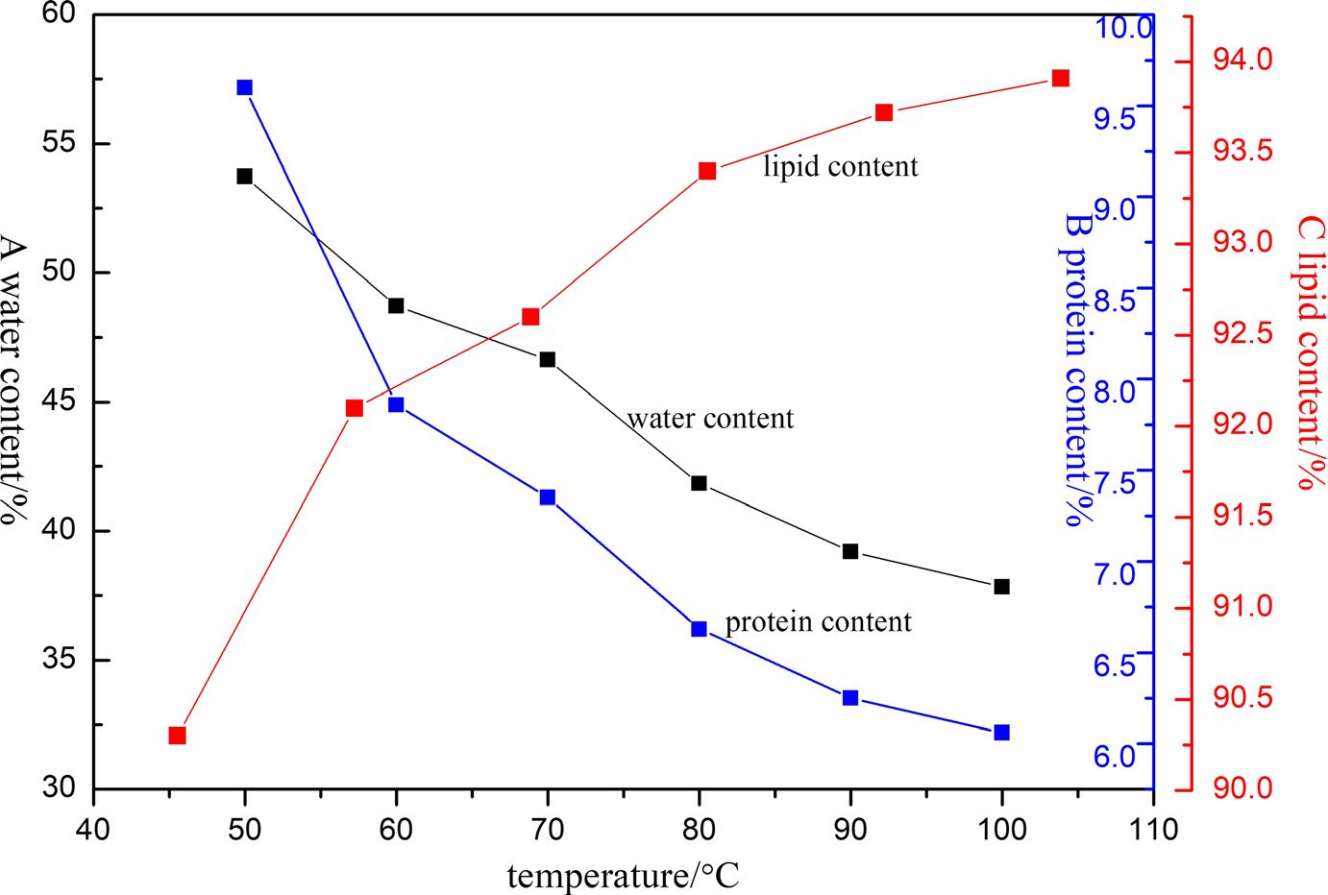
Effect of WBAE temperature on content of SOB enrichment

The SOB enrichment extracted at 4°C was used as a control group to explore the changes of the main components of the SOB enrichment extracted by WBAE at different temperatures, including changes in the contents of water, lipid, and protein. The results are shown in Figure 1. Because the source of raw materials and the method of extracting oil bodies were different, the content of SOB enrichment components determined may be slightly different from the results of other researchers (Ding et al., 2020; Zhao et al., 2016).

#### 3.1.1 Effect of WBAE temperature on moisture content of SOB enrichment

As shown in Figure 1a, the moisture content in the SOB enrichment decreased significantly with increasing the WBAE temperature. The SOB enrichment extracted from the control group (raw soymilk) contained the highest moisture content (53.73%, w/w). In this study, SOB enrichments were extracted from the aqueous phase by high‐speed centrifugation. It was considered that the moisture is the highest content than other components in the SOB enrichment. The protein component has a certain holding water capacity (Ainis et al., 2019). After heat treatment of raw soymilk, the exogenous proteins on the surface of the SOB were denatured in different degrees, and some exogenous proteins released from oil bodies induced by thermal treatment (Shen et al., 2012). The holding water capacity of the SOB enrichment decreases due to the release of exogenous proteins, which ultimately leads to the decrease of moisture content. With increasing the WBAE temperature, denaturation degree of exogenous proteins increased, and more exogenous proteins released from SOBs. This could explain the decreased moisture content in the order of 50, 60, 70, 80, 90, and 100°C. When the WBAE temperature is 100°C, the moisture content in the SOB enrichment reaches the lowest value of 37.83% (w/w).

#### 3.1.2 Effect of WBAE temperature on protein content of SOB enrichment

The protein content of SOB enrichment (dry basis) in the control group was 9.60% (w/w), and the protein content decreased significantly with increasing the temperature of WBAE. The proteins bound to SOB enrichment are mainly composed of two parts: the structural protein embedded in the SOB membrane and the exogenous protein attached to the surface of the SOB membrane during the extraction process (Zhao, Chen, Cao, et al., 2013; Zhao, Chen, Zhang, et al., 2013). The thermal stability of proteins is poor, and denaturation occurs at higher temperatures. Therefore, the higher temperature leaded to the higher denaturation degree of the exogenous proteins and the higher release degree of the exogenous proteins from the oil body (Wan et al., 2018). It has been reported that 90% of the exogenous protein will be released from the surface of SOB after the heat treatment of raw soymilk above 80°C (Shen et al., 2012). It can also be seen from Figure 1b that the protein content of the SOB enrichment is less than 6.63% (w/w) as WBAE temperature is above 80°C, and the protein content of SOB extracted at 100°C. It was indicated that the purity of SOB was higher with increasing the extracted temperature.

#### 3.1.3 Effect of WBAE temperature on lipid content of SOB enrichment

The lipid substance in SOB enrichment is mainly triglyceride, and there is also a small amount of phospholipid (the main component of the oil body membrane). As shown in Figure 1c, the lipid content of the SOB enrichment (dry basis) from the control group was 90.30% (w/w), and the lipid content increased significantly with increasing the WBAE temperature. The reason was that the protein content in the SOB enrichment decreased, and the purity of the SOB increased with increasing the WBAE temperature. Above 80°C, the rising trend of lipid content was gradually slowly, and the lipid content in the SOB enrichment extracted at 100°C reached the highest value (93.91%, w/w).

The results above revealed that the content of water, protein, and lipid in SOB enrichment was obviously changed with the increase of WBAE temperature. As the WBAE temperature increased, the purity of the SOB continued to rise. When the WBAE temperature was 100°C, a lot of the exogenous proteins bound to the SOB enrichment denatured and released from SOB. Higher purity SOB could be obtained by increasing the WBAE temperature.

#### 3.1.4 Effect of WBAE temperature on protein components in SOB enrichment

**FIGURE 2:**
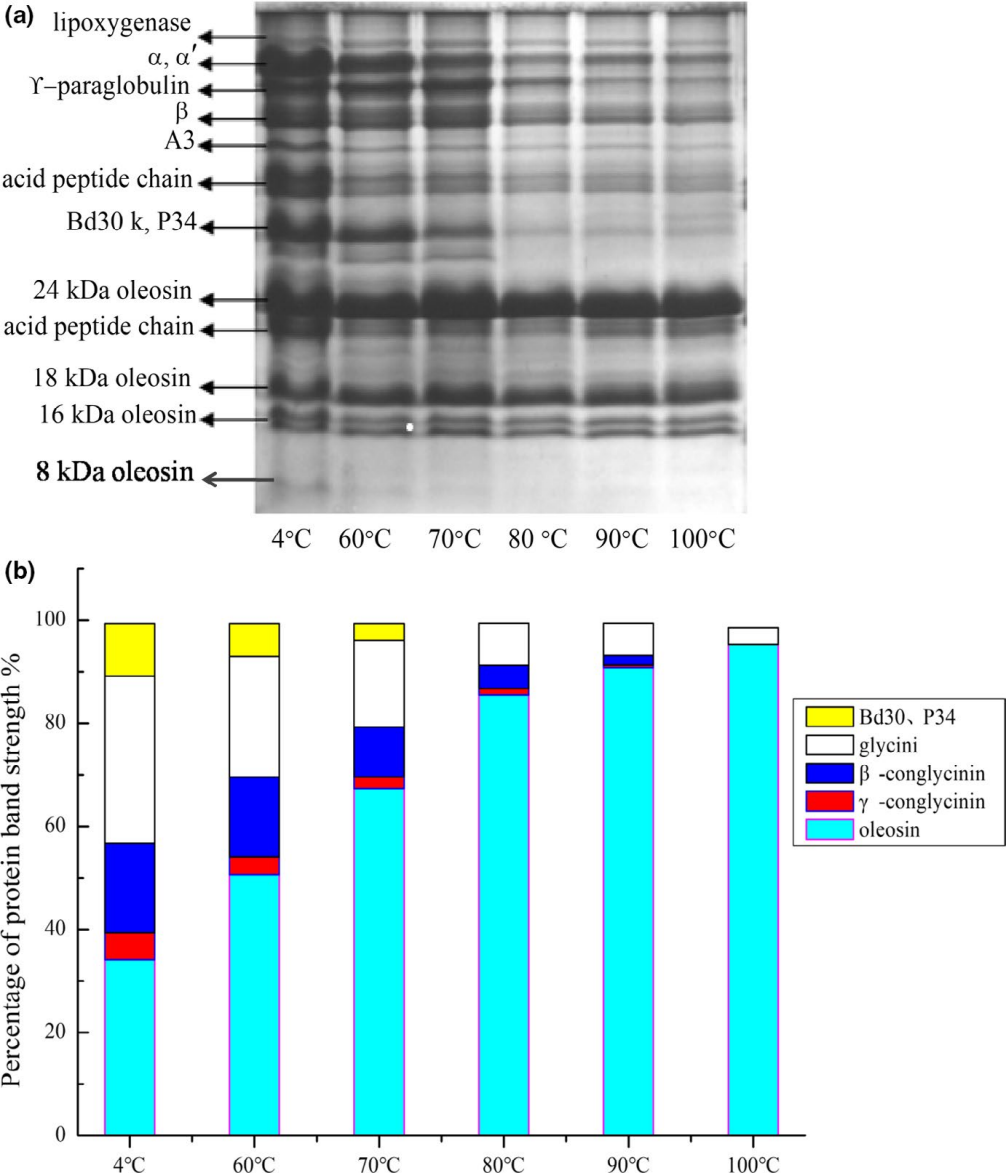
Effect of WBAE temperature on protein components in SOB enrichment. (a) Tricine‐SDS‐PAGE electrophoresis, (b) Protein band intensity percentage graph

The proteins in SOB enrichment mainly included structural proteins and exogenous proteins. The highest content of structural proteins included 24 kDa oleosin and 18 kDa oleosin. Exogenous proteins include lipoxygenase and glycinin (A_3_, acidic peptide chain, basic peptide chain), β‐conglobulin (α, α', and β), γ‐conglobulin, Bd 30K, and P34 (Chen, Cao et al., 2014; Chen, Zhao et al., 2014). It was reported that P34 was an protease that had proteolytic activity and high cleavage site specificity (Lys‐Thr of 24 kDa oleosin) (Zhao et al., 2017). Some researches showed that endogenous protease P34 of SOB enrichment extracted at the conditions of pH 6.5, room temperature, and water phase could hydrolyze 24 kDa oleosin to peptides (16 kDa and 8 kDa) within 1 hr (Zhao, Chen, Cao, et al., 2013; Zhao, Chen, Zhang, et al., 2013). Figure 2a showed that SOB enrichment from the control group contained 16 kDa and 8 kDa peptides, indicating that some 24 kDa oleosin had hydrolyzed before heating raw soymilk. However, the content of 24 kDa oleosin and 16 kDa (containing 16 kDa oleosin and 16 kDa peptide) in SOB enrichment did not unchange when the WBAE temperature was 60°C and above 60°C. It was attributed that 60°C and above 60°C could denature endogenous protease P34, and the hydrolysis of 24 kDa oleosin was inhibited, that is 16 kDa peptide did not change significantly (Zhao, Chen, Cao, et al., 2013; Zhao, Chen, Zhang, et al., 2013). In addition, the electrophoretic gel also showed that protein band intensity of other structural proteins (18 kDa oleosin and 16 kDa oleosin) did not change at the whole process of WBAE, that is, structural proteins were not hydrolyzed significantly with increasing the WBAE temperature. However, the protein band intensity of the exogenous protein was gradually weakened with the increase of the WBAE temperature, indicating that the absolute content of the exogenous protein gradually reduced.

As shown in Figure 2b, the percent strength of the structural proteins in the SOB enrichment from control group was 34.06%, and the exogenous proteins were 65.29%. With increasing the WBAE temperature, the band intensity percentage of structural proteins gradually increased, while the band intensity percentage of exogenous proteins gradually decreased. The change trends of both structural proteins and exogenous proteins were significant (*p* < .05), which indicated that the changes of relative content for structural and exogenous proteins were significantly. The exogenous proteins were the main proteins of SOB enrichment extracted at 4°C, while the structural proteins gradually became the main proteins with increasing the WBAE temperature. For the SOB enrichment extracted at 100°C, the band strength percentage of structural proteins was 95.26%. The reason for this change was that when the soymilk was heated, the exogenous proteins would denatured and released from SOBs with the increase of temperature. When the extraction temperature was 100°C, the purity of the obtained SOB is relatively higher, and the content of structural proteins was above 90%.

Therefore, the WBAE temperature affected the components of exogenous protein in SOB enrichments, and high temperature inhibited the hydrolysis of structural proteins, which could maintain the integrity of SOB structure.

### 3.2 The effect of WBAE temperature on the properties of SOB emulsion

#### 3.2.1 Effect of WBAE temperature on zeta potential of SOB emulsion

**FIGURE 3:**
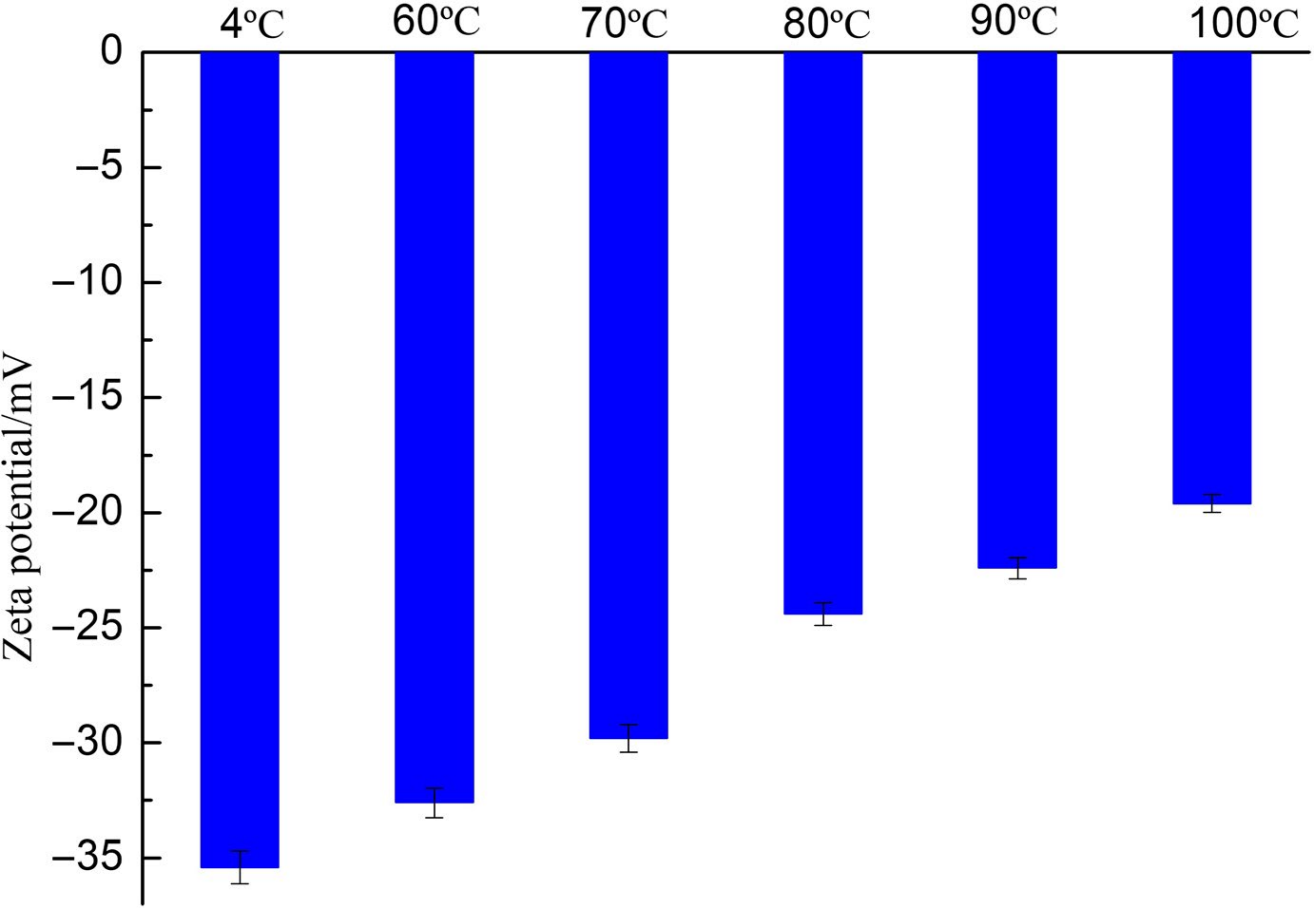
Effect of WBAE temperature on zeta potential of soybean body emulsion

The dispersion or aggregation state of SOB emulsion was related to the electrostatic repulsion among the droplets. The charge of the droplet determined the electrostatic repulsion, which was usually reflected by the zeta potential. As shown in Figure 3, the zeta potentials of all SOB emulsions were negative, and it could be seen that the charges carried by the droplets in each group were negative. The zeta potential of the SOB emulsion from the control group was −35.4 mV, and the zeta potential of the SOB emulsion extracted at from 100°C was −19.6 mV. With increasing the WBAE temperature, the absolute value of the zeta potential of the emulsion significantly reduced, reflecting that the amount of charge carried by the droplets in the emulsion gradually reduced. The reason might be related to the denaturation and release of exogenous proteins (Yan et al., 2016). It was attributed that heat treatment leaded to the denaturation of exogenous proteins bound to the SOB surface, and the amount of exogenous proteins was released from the SOB. And the isoelectric points of glycinin, β‐conglycinin, and P34 were pH 4.5, 4.9–5.2, and 4.7–5.0, respectively. This could be used for explaining the weakening force among each SOB. As the WBAE temperature increased, the charge density on the surface of SOB and the zeta potential of the SOB emulsion also decreased. In this case, the droplets might be aggregate.

#### 3.2.2 Effect of WBAE temperature on the average particle size of SOB emulsion

**FIGURE 4:**
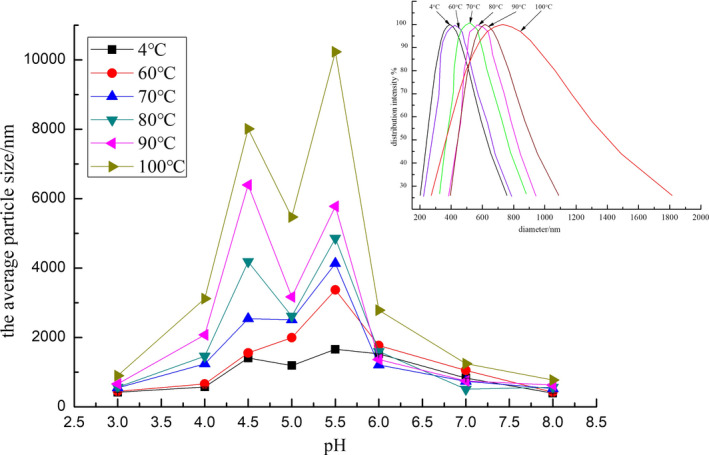
Effect of WBAE temperature on the average particle size of soybean body emulsion and particle size distribution of soybean body emulsion (pH 8)

The average particle size of SOB emulsion directly reflected the dispersibility of droplets in emulsion as shown in Figure 4. With increasing pH value from 3 to 8, the average particle size of all SOB emulsion increased gradually and then decreased. It was attributed that the pH value of SOB emulsions was less than the isoelectric point of the proteins, and the negative charges carried by the droplets in the emulsion gradually decreased, and the electrostatic repulsion between the droplets decreased (Kulmyrzaev et al., 2000). The aggregation of SOBs occurred due to the weak electrostatic repulsion near the isoelectric point of proteins. In contrast, SOBs tended to be dispersed as individuals, when the pH value of SOB emulsion was higher than the isoelectric point of the proteins bound to SOB. As the degree of droplet aggregation decreased, the average particle size of the emulsion gradually decreased.

As shown in Figure 4, the average particle size of the SOB emulsion also gradually increased at each pH value with increasing the WBAE temperature. At the pH 5.5, the average particle sizes of SOBs extracted at 60, 70, 80, 90, and 100°C were 3.2, 4.05, 4.97, 5.2, and 10.23 μm, respectively. Figure 3 showed that the charge of SOB decreased with the increasing the WBAE temperature. In other words, the electrostatic repulsion between the droplets in the emulsion decreased. Therefore, the aggregation degree of the droplets increased, resulting in increasing the average particle size.

Particle size distributions of SOBs extracted at 60, 70, 80, 90, and 100°C were analyzed at pH 8.0, which could prevent the aggregation of SOBs. As shown in Figure 4, all SOB emulsions showed a monomodal distribution at pH 8.0. It was considered that the physical stability of the SOB emulsions was good at pH 8.0. In addition, the most of droplets exist in the form of individual rather than aggregates, and the average particle size of the emulsion at pH 8.0 reflected the true size of the particles in the SOB emulsions. At pH 8, the particle size of the SOB emulsion from control group was distributed from 201.7 nm to 757.5 nm. With the increase of WBAE temperature, the particle size and distribution range of emulsions gradually increased. The particle sizes of the SOBs extracted at 60 ~ 100°C were mainly distributed between 325.3 ~ 1825.9 nm. It was considered that the increase of particle size was not only the droplet aggregation caused by the decrease of electrostatic repulsion force, but also the coalescence of droplets. It was reported that the more extrinsic proteins were bound to SOBs, the more easily the heat‐induced coalescence of SOBs occurred (Zhao et al., 2016). According to Figure 2, the exogenous proteins bound to SOBs decreased with increasing the WBAE temperature. These results showed that the WBAE temperature affected the particle size of SOBs and physical stability due to the changes of exogenous proteins and the interfacial properties of SOBs.

**FIGURE 5:**
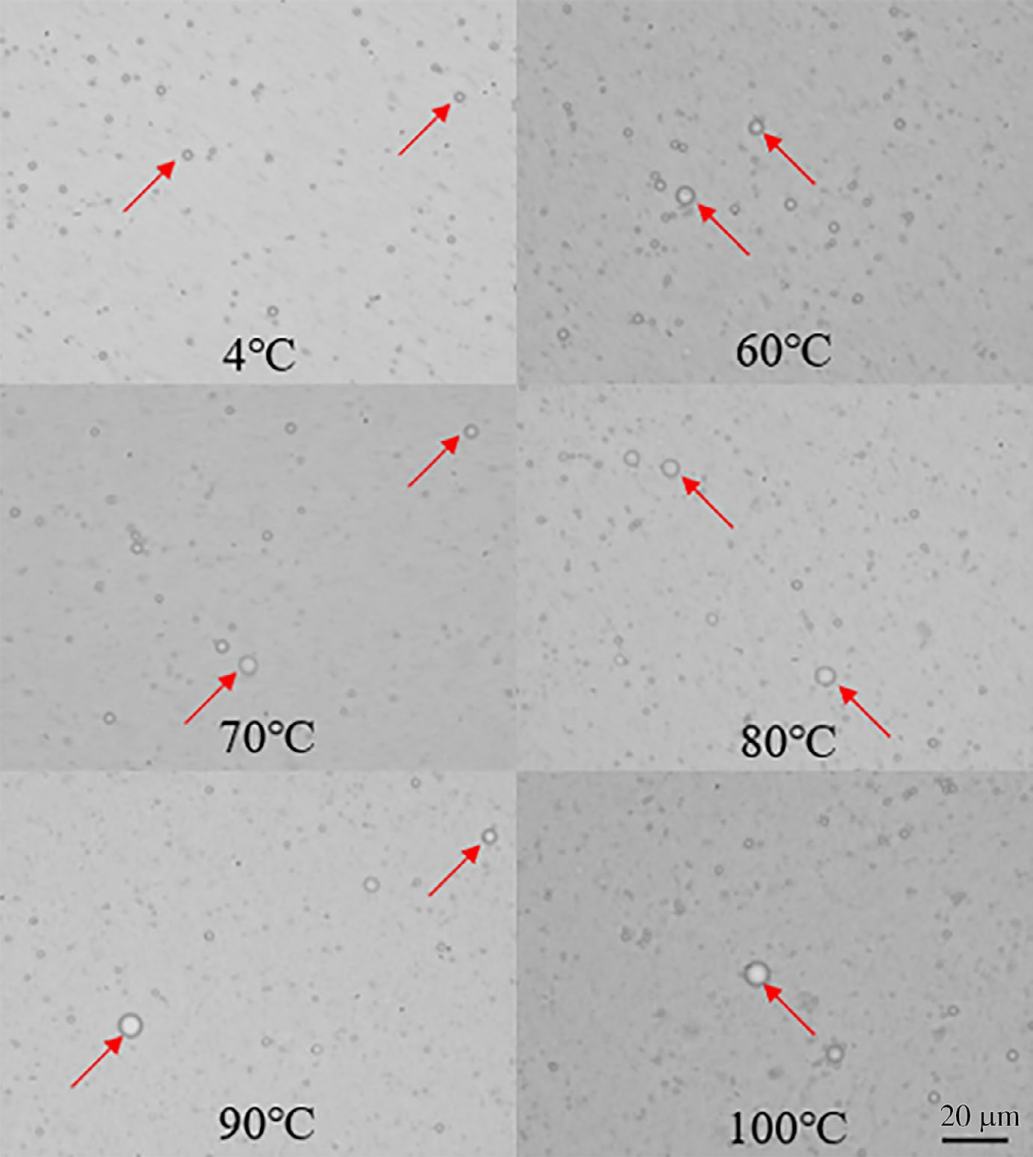
Effect of WBAE temperature on SOB fusion

In order to verify the coalescence among SOBs during hot extraction, all SOB emulsions were diluted and observed with a fluorescent inverted microscope. The results were shown in Figure 5. It can be seen that the control group had the smallest droplets in the SOB emulsion, and few droplets were observed. However, the SOBs extracted at 60, 70, 80, 90, and 100°C had some large diameter droplets, and with the increase of the WBAE temperature, the droplets diameter of SOBs gradually increased. The droplet diameter of SOB extracted at 100°C was about 4 μm. This result also verified that the different degrees of coalescence among the SOBs leaded to the increase of particle size. By heating, the exogenous proteins bound to SOBs were denatured, and exogenous proteins exposed the hydrophobic groups, and the coalescence of OBs occurred by hydrophobic interaction (Nikiforidis et al., 2011). With increasing the WBAE temperature, the more easily the heat‐induced coalescence of OBs occurred, the larger diameter of the droplets produced.

#### 3.2.3 Effect of WBAE temperature on oxidation stability of SOB emulsion

**FIGURE 6:**
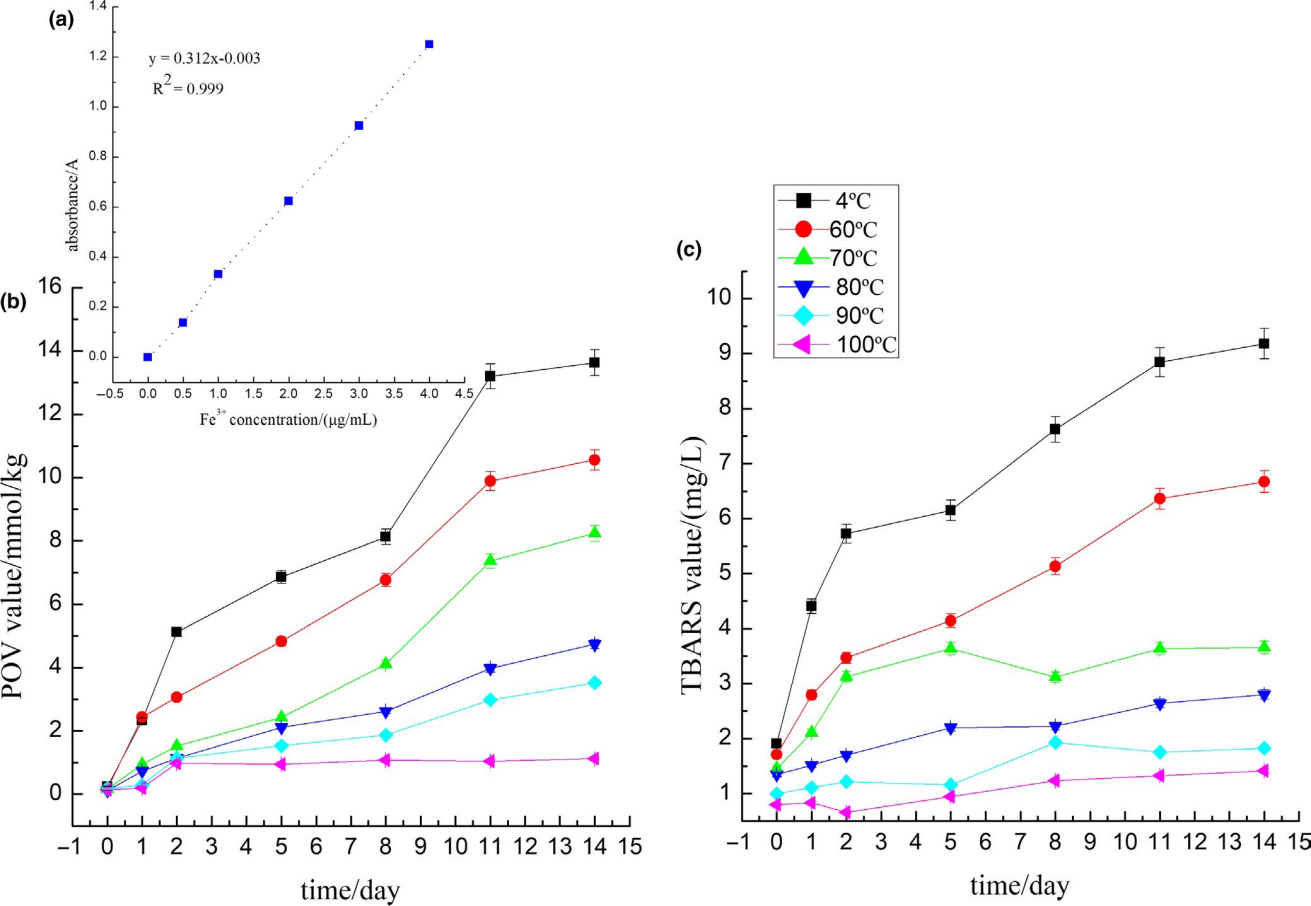
Effect of WBAE temperature on oxidation stability of SOB emulsion. (a) Fe^3+^ standard curve, (b) POV value, (c) TBARS value

Figure 6a showed the standard curve of Fe^3+^. According to the concentration of Fe^3+^ (μg/ml) and the absorbance measured at 510 nm, the regression equation (y = 0.3126x–0.0031, *R* ^2^ = 0.9994) was obtained. The results showed that Fe^3+^ had a good linear relationship in the concentration range of 0 ~ 4 μg/ml. During storage, the lipid oxidation of SOB emulsions produced hydroperoxide and malondialdehyde. The POV value reflected the hydroperoxides (primary products) content, and the TBARS value reflected the malondialdehyde (secondary products) content in the SOB emulsions. As shown in Figure 6b,c, the POV and TBARS values of SOB emulsions extracted at 4°C, 60°C, and 70°C showed an upward trend with the prolonged storage time. It was attributed that endogenous protease P34 bound to SOBs could hydrolyze the structural proteins, resulting in the enzymatic oxidation. Zhao et al. (2016) reported SOBs extracted from raw soymilk (pH 6.8) contained the proteases P34, lipoxygenase, and phospholipidase D. High temperature could denature these enzymes. When the WBAE temperature was above 60°C, P34 was gradually inactivated, and the hydrolysis of structural proteins induced by P34 was inhibited, which decreased the enzymatic oxidation of triglycerides. Therefore, the POV and TBARS values of SOBs extracted at 60°C and 70°C were lower than the control group during storage. With increasing the WBAE temperature from 80°C to 100°C, the POV and TBARS values of SOB emulsions increased slowly with prolonged storage days at similar rates. Moreover, the POV and TBRAS values were constantly lower in SOB extracted at 100°C compared to other SOB emulsions during the storage time. This might be correlated with the proteases P34, lipoxygenase, and phospholipidase D. The oxidative stability of the SOB extracted at 100°C was greatly improved, which was attributed to the inactivation of the enzymes above (Chen, Cao et al., 2014; Chen, Zhao et al., 2014). In other words, the lipids of SOB extracted at 100°C were oxidized automatically rather than enzymatic oxidation, and the oxidation rate of the lipid was low. Therefore, the content of oxidation products kept at a low level. The results above revealed that the oxidation of lipids was obviously affected by the WBAE temperature, and the oxidative stability of the SOB was improved by increasing the WBAE temperature.

#### 3.2.4 Effect of WBAE temperature on viscosity characteristics of SOB emulsion

**FIGURE 7:**
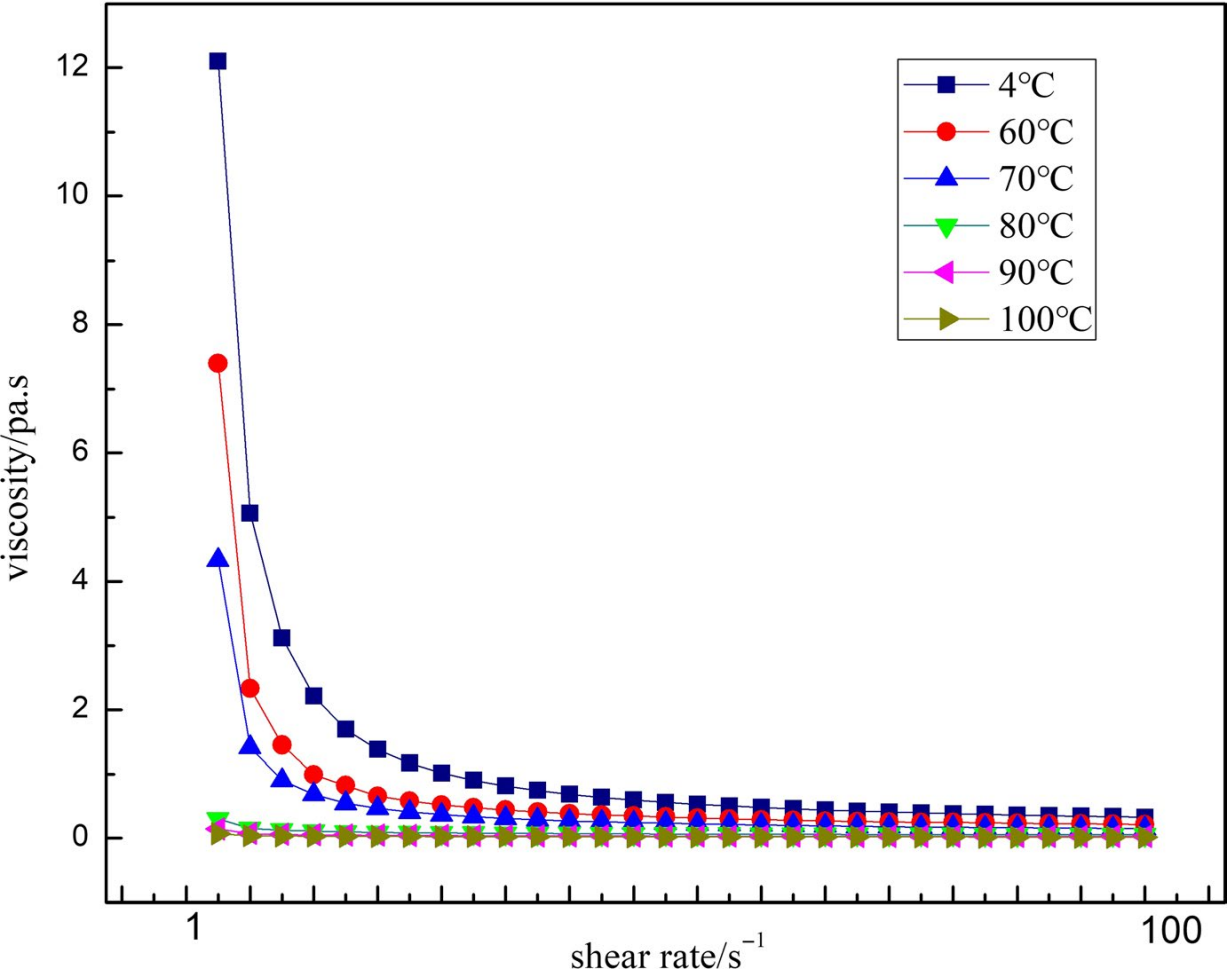
Effect of WBAE temperature on viscosity characteristics of SOB emulsion

The preparation of SOB emulsion was different from protein‐stabilized emulsion, and SOB emulsion prepared was not homogenized. However, SOB emulsion was similar to protein‐stabilized emulsion, and their shear thinning characteristics were caused by droplet flocculation (Floury et al., 2000). As shown in Figure 7 and Table 1, the viscosity of all SOB emulsions decreased with increasing shear rate, which showed shear thinning behavior. when the shear rate was low, the small shear force could not destroy the interaction between the droplets in the emulsion at the condition of low shear rate. As the shear rate increased, the droplet aggregates were destroyed, the viscosity of the emulsion decreased (Cao et al., 2015). Figure 6 also showed that the viscosity of the SOB emulsions decreased with the increase of the WBAE temperature, and the viscosity of the emulsion decreases significantly with the increase of the temperature (*p* < .05) with the WBAE temperature increasing from 4 to 80°C. However, when the WBAE temperature was above 80°C, the viscosity of SOB no longer decreased significantly (*p* > .05). The reason was that the exogenous proteins of the SOBs were denatured and released with the increase of the WBAE temperature. After SOBs were prepared to the emulsion, the interaction among the droplets was weaken. As a result, the viscosity of the SOB emulsion reduced. The exogenous protein contents of SOB extracted at 80–100°C was less than 15% (Figure 2), and the decrease of emulsion viscosity was not significant. Nikiforidis et al. (2011) had reported similar results, that is, the viscosity of corn germ oil body emulsion decreased with decreasing the exogenous protein content.

**TABLE 1:** Effect of WBAE temperature on fluidity index (n) and consistency coefficient (k) of SOB emulsion

Items	WBAE temperature					
	4°C	60°C	70°C	80°C	90°C	100°C
*n* value	0.16 ± 0.02^a^	0.28 ± 0.05 ^b^	0.47 ± 0.07 ^c^	0.65 ± 0.09 ^d^	0.70 ± 0.05 ^e^	0.73 ± 0.04 ^e^
k value (Pa•s)	1.51 ± 0.12^a^	0.76 ± 0.09^b^	0.40 ± 0.08^c^	0.13 ± 0.02^d^	0.06 ± 0.01^e^	0.05 ± 0.01^e^

Note

Different letters in the same row represent significant differences (*p* < .05).

In addition, Table 1 showed that the consistency coefficient (k value) of the control group was 1.51, and the k value of other experimental groups decreased significantly with the increase of temperature. It indicated that increasing the WBAE temperature could improve the fluidity of the SOB emulsion, and SOB extracted by WBAE could be used for the development of liquid foods, such as beverages.

## 4 CONCLUSIONS

Soybean oil body enrichments obtained by WBAE at different temperatures had different components, and the physicochemical properties of SOB emulsions were different. The WBAE temperature mainly affected the exogenous proteins bound to SOB, and the exogenous protein was gradually denatured and released from SOBs. Therefore, the purity of SOB increased with the WBAE temperature. In addition, the protein and moisture content decreased, and the lipid content increased with increasing the WBAE temperature. The physical and chemical properties of SOB emulsions were affected by the WBAE temperature. As the WBAE temperature increase, the zeta potential and viscosity of the SOB emulsion were decreased, while the oxidative stability was improved. By changing the extraction temperature of SOB, the SOBs with different components and physicochemical properties were obtained. The SOB extracted at 100°C had better purity, and the oxidation stability and fluidity were better than other SOB. The SOB extracted at 100°C had excellent potential for developing liquid food.

## CONFLICTS OF INTEREST

The authors declare no conflict of interest.
